# The land of homesickness: The impact of homesteads on the social integration of rural migrants

**DOI:** 10.1371/journal.pone.0307605

**Published:** 2024-07-25

**Authors:** Yanbo Wu, Minghao Xu, Shichen Li, Hongwei Wang, Qi Dong

**Affiliations:** 1 School of Public Economics and Administration, Shanghai University of Finance and Economics, Shanghai, China; 2 School of Finance and Economics, Henan Polytechnic, Zhengzhou, China; 3 School of Business, Anhui University of Technology, Ma’anshan, China; 4 College of Business, Shanghai University of Finance and Economics, Shanghai, China; Tallinn University of Technology School of Engineering: Tallinna Tehnikaulikool Inseneriteaduskond, ESTONIA

## Abstract

Promoting the social integration of rural migrants is key to improving the mechanism of rural-urban integration and development. This study utilizes the 2017 China Migrants Dynamic Survey and matched urban macro data to systematically explore the impact of homesteads on the social integration of rural migrants. Research has shown that social integration of rural migrants will be inhibited if they own homesteads. Simultaneously, the degree of inhibition varies according to the individual characteristics of rural migrants, the region to which they belong, and other factors. Specifically, when rural migrants aged 18 to 50 own homesteads, their degree of social integration into the cities they move into will be low. At the same time, for rural migrants in the central region, homestead ownership will not affect their degree of social integration. In addition, the mechanism analysis shows that increased housing expenditure inhibits rural migrants’ willingness to integrate. Meanwhile, owning contracted land and owning a house in the city also affect the degree of social integration of rural migrants to a certain extent. The findings of this study can broaden research on the effects of land on the free movement of population factors. In the meantime, it provides theoretical references for improving the level of social integration of migrants, enhancing people’s well-being, and improving the mechanism of urban-rural integration and development.

## 1. Introduction

Since its reform and opening-up, China has experienced an unprecedented urban revolution. An important feature of this revolution is the large-scale migration of people to cities [1]. According to China’s National Bureau of Statistics, the urbanization level of China’s permanent resident population reached 64.72% in 2021, and the migrant population reached 376 million, accounting for 26% of the country’s total population. Most migrants move for business or work, and this mobility fills the gap in the urban labor force and contributes significantly to economic development. According to the Rostovian take-off model [2], China is in the late stage of the take-off phase and the early stage towards maturity. However, objectively speaking, the urbanization rate of China’s household registered population is only 45.4%, which is far behind the urbanization rate of the permanent resident population. The gap between the urbanization rate of the permanent resident population and the urbanization rate of the household registered population indicates that China’s new urbanization process has a long way to go. The root cause is that a large number of rural migrants are still facing the dilemma of "not being able to go back to the countryside and not being able to integrate into the city" and the characteristic of "semi-urbanization" is still relatively obvious [3]. Promoting the transformation of rural migrant workers into urban residents and providing migrant populations with equal access to basic public services have become the key tasks of China’s new urbanization. Realizing this goal requires not only the support and guidance of relevant government policies but also the migrant population’s strong willingness to take up long-term residence in the destination city [4]. Therefore, understanding the willingness of the migrant population to take up long-term residence and exploring its determinants has become a major issue that needs to be urgently addressed in the process of modernization in China.

From the perspective of residence, "living and working in peace and contentment" has always been a cultural tradition and a good wish for Chinese people. This concept has profoundly influenced contemporary residents’ housing choices and social behaviors. Solving the integration problems of the migrant population is a prerequisite for their survival and long-term residence in the inflow area as well as a basic guarantee to promote their full integration into the inflow city [5]. In recent years, the migrant population has begun to undergo a major transformation, manifesting itself in more stable employment and longer residence in destination cities [6]. Fundamentally, large-scale population mobility is an inevitable result of socioeconomic transformation and upgrading [7], and the realization of "a place to live" is a common aspiration. However, owing to the relatively high level of urban housing prices [8], it is difficult for the migrant population to obtain house ownership. This also makes them significantly more mobile than local residents and they experience more housing instability [4]. All kinds of shackles require the migrant population to live on the edge of the city and change their place of residence frequently. Undoubtedly, this frequent mobility behavior will cause them to lack a sense of identity as local residents [9]. Therefore, housing is an important material basis for migrant populations to live in a city for a long time, and purchasing a new house in the city is seen as one of the hallmarks of success [10]. Due to China’s special national conditions, rural migrants tend to take land in their hometowns as their last resort. At the same time, they must keep in touch with their inflow cities by purchasing housing [11]. Therefore, at this level, whether rural migrants have land security in their hometowns may affect their degree of integration in cities. The existing literature tends to focus more on individual characteristic factors when studying the willingness of migrants to settle [12, 13]. Only a few scholars have studied the impact of rural land on migrant populations [14]. Therefore, it is of great practical significance to study the impact of homesteads on the urban integration of rural migrant populations.

Promoting the social integration of migrant populations is key to influencing people-centered urbanization. This social integration is slightly different from population migration, with the main emphasis on the attractiveness of the city of inflow and emotional decision-making issues of the migrant population. Factors affecting social integration include not only the individual’s psychological satisfaction with life, but also the family’s future prospects. Of course, the environment in which migrants live and related policies also affect their sense of integration into the city [15]. From the perspective of social exclusion as non-institutional ownership, social exclusion presents itself in the form of segregation. At the same time, segregation is not limited to physical space but also includes social relations and networks [16]. The segregation of social relations and networks amplifies the dominant position of the dominant group in society, thus reinforcing the unequal distribution of social resources. This phenomenon tends to create a sense of psychological deprivation among vulnerable groups, mainly migrant workers, which affects their level of social integration [17]. Therefore, a good community economic environment and convenient services can promote the economic integration of migrants into the city of inflow and enhance their social adaptation. Undeniably, when the degree of social adaptation of the migrant population increases, they will be more likely to move from segregation to rationality and compatibility, which in turn will enhance their willingness to integrate [18].

## 2. Literature review and theoretical analysis

In recent years, the government has actively promoted the process of urbanization and formulated a series of policies for the citizenship of the rural migrant population. However, China’s urbanization process has not yet met expectations. An important reason for this is that most of these policies have centered on requiring migrants to give up their rural land, including contracted land and homesteads [19]. Land is a scarce resource, not only as a factor of production but also as a very important survival support function [20]. Contracted land has long been responsible for the livelihood and employment functions of the agricultural population, while homesteads have effectively guaranteed "a home for everyone" [21]. As the main body of collective rural construction land, the right to use homesteads allows farmers to enjoy building residences and directing domination. When rural migrants’ own homesteads, their perception of homesteads as a barrier is reinforced, thus consolidating their willingness to return to their hometowns [22]. However, with the increase in the proportion of non-agricultural income of migrant workers’ families and the "separation of people and houses" caused by population migration, the productive and secure functions of rural land may gradually weaken. With the continuous development of society and the implementation of land system reform, a vast number of migrant workers’ cognition of land has undergone significant changes. In the view of many migrant workers, land is no longer just a resource but also an asset [23]. The property value of land has become increasingly prominent. Some scholars have found that the willingness to integrate into the city and the likelihood of moving their household registration out of the countryside is lower for rural migrants with a homestead than for migrants without a homestead in the countryside [24]. However, legal disputes still exist regarding the rights of homesteads, and the property functions of homesteads are difficult to reflect. At the same time, the residential security function of the homestead provides psychological comfort to the migrant population [25], which inhibits their tendency to become citizens to a certain extent. Of course, other scholars hold opposing opinions. These scholars believe that homesteads not only have the effect of labor transfer but also of citizenship on labor allocation. In other words, homesteads can significantly enhance migrant workers’ willingness to become citizens [26]. With the promotion of a new round of land property rights policies, China is comprehensively resolving historical legacy issues and seeking to fundamentally safeguard the land interests of farmers, thereby reducing migrant populations’ concerns about losing their land.

Social integration is a classical sociological topic. This means that after the floating population flows into other cities, they begin to interact with local people in various aspects, such as economy and culture [27]. On this basis, they gradually identify with the customs, lifestyles or modes of doing things in the cities where they migrate. After identification, these migrant populations gradually change their behavior and eventually transform into an urban paradigm [11]. This process has clear dynamic and progressive characteristics. Improving access to resources for relatively disadvantaged groups is a key step in social integration. This can alleviate or even eliminate inequalities caused by social exclusion or segregation [28]. Therefore, promoting social integration is of great significance for building a harmonious society.

According to the classic push-and-pull model, the choice of migrants to integrate into the city or not is a rational weighing of the "cost-benefit" of entering the city and exiting the countryside [29]. The direction in which migrants ultimately choose to migrate depends on which factors dominate in the process of moving in and out. Specifically, from the perspective of the outflow area, factors such as deficiency of resource endowment, lower economic income level and development prospect are the unfavorable factors for migrants to live in the local area. These factors push the original residents out of the local area [30], so they are the "push" role in the process of population mobility [31]. The direction in which migrants ultimately choose to migrate depends on which factors dominate in the process of moving in and out [32]. Specifically, from the perspective of the outflow area, factors such as deficiency of resource endowment, lower economic income level and development prospect are the unfavorable factors for migrants to live in the local area [33]. These factors push the original residents out of the local area, so they are the "push" role in the process of population mobility.

As rational individuals, migrants tend to weigh their choices of giving up land and settling in cities in order to maximize their personal or family benefits [34]. Current scholars have mostly focused on impeding or facilitating factors at the urban level and the individual level of migrants. Comparatively, current research ignores the impact of rural land [35], and there is a particular lack of literature on how rural homesteads affect migrants’ willingness to integrate into cities. However, with the increasing national emphasis on rural areas, the improvement of rural infrastructure brought about by the construction of better villages and the effective management of rural poverty through poverty eradication, the gap between urban and rural areas has narrowed [36]. Farmers’ sense of well-being and acquisition is increasing day by day. Their incentive to leave the countryside has weakened. As an important part of rural culture, the homestead has deep cultural and emotional significance [37]. For most farmers with deep-rooted traditional concepts, the homestead not only carries their childhood memories, but is also a kind of symbol to maintain the family [34]. Many farmers even use the homestead as their "backing" to fight in the big city, providing them with a secure place to live and retire. Therefore, this concept of security and "local attachment" plot makes some rural migrants reluctant to leave their hometowns. This will lead to their lower willingness to stay in the city and weaker willingness to integrate. In other words, the homestead serves as a "pull” force from the place of departure for migrants.

Additionally, the value of urban household registration has declined significantly. In addition to the dichotomy that still exists in a few rights and interests, such as children’s education and social security, the transformation of peasants into citizens does not yield many additional social welfare benefits. This obviously weakens the willingness of migrant workers to enter the city. Of course, this is also the "push" force from the city of inflow. Existing literature suggests that the difference between urban residents and migrants is ultimately a function of house ownership [36]. When migrants own houses in their mobility destinations, it could be a direct reflection of their long-term residential willingness and social integration [38]. If their housing problem can be solved directly, it means that migrants can convert the expenditures they would have spent on renting a house into other disposable income. This promotes their willingness to reside in the long term economically, transforming the resistance of not owning a house into a “pull” force [39].

## 3. Research design

### 3.1 Data sources and selection notes

The data used in this study were obtained from the 2017 China Migrants Dynamic Survey (CMDS). The survey of this data covers 31 provinces (autonomous regions or municipalities directly under the Central Government) and Xinjiang Construction Corps in the country where the inflow of migrants is more concentrated. Data sampling was stratified, multistage, and proportional to size. At the same time, the data are based on migrants who have lived in the local area for more than one month, who are not household members of the region (county or city), and who are aged 15 years and above. The sample size was large and representative.

Before proceeding to the empirical study, the following treatments were applied to the sample. First, to alleviate the research bias caused by the age of the sample, this study limited the age of the sample to 18–65 years old. Second, because the research object of this study is the rural migrant population, it restricts the household registration of the sample. This is done by retaining only samples whose household registration is agriculture or whose household location is rural. Third, considering that the ownership of outliers may bias the research conclusions of this study, this study shrinks the outliers of continuous variables that exceed the reasonable range in the sample data. Meanwhile, to ensure the completeness of the data, missing and invalid samples were eliminated. Fourth, to make the empirical results of this study represent the overall situation as much as possible, the sample data of cities with individual sample sizes less than 200 are excluded from the calculation. After the sample was preprocessed, 68817 valid observations were obtained.

### 3.2 Empirical strategy and variable definition

This study examined the impact of homesteads on the social integration of rural migrants. Therefore, the following econometric model was constructed to conduct the regression in this study:

$$Integration_{ij}=\alpha +\beta \;zhaijidi_{ij}+\lambda_{1}I_{ij}+\lambda_{2}F_{ij}+\lambda_{3}M_{ij}+\theta_{j}+\varepsilon_{ij}$$ (1)

In this model, *Integration* is the explained variable representing the degree of social integration among the rural migrant population. *zhaijidi* denotes whether the rural migrant population owns a homestead, which serves as the core explanatory variable. I,F and M represent the set of control variables for the dimensions of individual, family, and migration characteristics. The subscripts i and j denote individual respondent households as well as the place of inflow of respondent households. The θ is the city fixed effect. The ε_*ij*_ is a random disturbance term.

#### 3.2.1 Explained variable: Degree of social integration

Based on the relevant practices of existing scholars, this study calculated the index of social integration through a comprehensive calculation of eight questions (Zhang Yalin). The questions are "I like the city/place where I live now" "I pay attention to the changes in the city/place where I live now" "I would like to integrate and be part of the local people" "I feel that locals are willing to accept me as one of them" "I feel that locals look down on outsiders" "It is more important for me to follow the customs of my hometown" "There is a big difference between my hygiene habits and those of local citizens" "I feel that I am already a local" Specifically, on the basis of standardizing the eight variables, indicators whose cumulative contribution rate is at or above 85% are selected through principal component analysis. Based on this, the comprehensive score for the degree of social integration of each rural migrant population was calculated.

#### 3.2.2 Core explanatory variable: Ownership of a homestead

This variable is based on the value of the question "Do you have a homestead in your hometown". To make the final conclusions of the study more accurate, this paper excludes the samples that answered "I don’t know" The option of "yes" is assigned a value of 1, and the option of "no" is assigned a value of 0.

#### 3.2.3 Control variables

The control variables were divided into three categories: individual, household, and migration characteristics. Individual characteristics included gender, age, age squared/1000, education level, ethnicity, political profile, marital status, and health status. Household characteristics include monthly household income, household expenditure, and household size. The migration characteristics include the time of mobility, number of cities of mobility, and scope of mobility. Table 1 lists the main variables involved in the empirical process and their meanings.

**Table 1:** Variable definition.

Dimension	Variable Name	Variable Definition
explained variable	Degree of social integration	Degree of social integration of respondents. The higher the value, the higher the degree of integration.
explanatory variable	Ownership of a homestead	Respondent has a homestead in his/her hometown (household registration) = 1; otherwise = 0
individual characteristics	Gender	Male = 1; Female = 0
	Age	Age of respondents in years
	Age^2^/1000	age^2^/1000
	Marriage	First marriage or remarriage = 1; otherwise = 0
	Nation	Han Chinese = 1; Otherwise = 0
	Education	No schooling = 1; Elementary school = 2; Junior high school = 3; High school/technical secondary school = 4; junior college = 5; Undergraduate college = 6; Postgraduate students = 7
	Health	Can’t look after themselves = 1; unhealthy, but can able to take care of themselves = 2; Basically healthy = 3; healthy = 4
	Political profile	Respondent is a member of the Communist Party of China = 1; otherwise = 0
household characteristics	Monthly household income	Respondent’s total monthly household income
	Monthly household expenditure	Respondent’s total monthly household expenditure
	Size of family members	Number of family members living together
migration characteristics	Duration of migration	Duration of respondent’s current migration
	Cities of migration	How many cities (including current residence) have respondents moved to (in)
	Range of migration	range of respondents’ current migration: across provinces = 1; across cities within provinces = 2; across counties within cities = 3

### 3.3 Descriptive statistics of variables

Table 2 presents descriptive statistics for the main variables. Among the 68817 valid survey samples, the average value of the social integration degree of the rural migrant population is approximately 3.458, and the difference between the minimum and maximum values is 4.405, indicating that the degree of integration of different rural migrant populations for the city of inflow varies greatly. Meanwhile, about 76% of the rural migrant population owns a homestead. Therefore, it is important to explore the impact of the presence or absence of a homestead on the degree of social integration of the rural migrant population. The average age of the rural migrant population in this study is about 36 years, 82% of the migrant population is married, the average monthly household income is 6951 yuan, the average monthly household expenditure is 3482 yuan, the average duration of migration is 6 years, and the longest duration of migration of the rural migrant population reaches 46 years.

**Table 2:** Descriptive statistics.

dimension	variable name	Mean	St.D	Min	P25	P50	P75	Max
explained variable	Degree of social integration	3.458	0.741	0.0940	3.031	3.031	4.137	4.50
explanatory variable	Ownership of a homestead	0.758	0.428	0	1	1	1	1
individual characteristics	Gender	0.587	0.492	0	0	1	1	1
	Age	36.29	9.724	18	29	35	44	65
	Age^2^/1000	1.411	0.747	0.324	0.841	1.225	1.936	4.23
	Education	3.250	1.010	1	3	3	4	7
	nation	0.912	0.283	0	1	1	1	1
	Political profile	0.0330	0.177	0	0	0	0	1
	Marriage	0.822	0.382	0	0	1	1	1
	Health	3.832	0.411	1	1	4	4	4
household characteristics	Monthly household income	6951	4421	1500	4000	6000	8000	30000
	Monthly household expenditure	3482	2170	500	2000	3000	4100	13,000
	Size of family members	3.185	1.187	1	3	3	4	10
migration characteristics	Duration of migration	6.226	5.982	0	2	4	9	46
	Cities of migration	2.066	1.968	1	1	2	2	30
	Range of migration	2.403	0.716	1	2	3	3	3

## 4. Empirical test

### 4.1 Regression analysis

Table 3 shows the regression results of the impact of owning homestead on the social integration of residents. Column (1) of Table 3 reports the regression results of the effect of owning a homestead on the degree of social integration. The results show that rural migrants who own homesteads have a lower degree of social integration than those who do not own homesteads. This effect is statistically significant at the 1% level. Then, the control variables are added step-by-step. Column (2) adds control variables for individual characteristics, Column (3) adds control variables for household characteristics, and Column (4) adds control variables for migration characteristics. According to the regression results in these columns, the coefficient estimates of the explanatory variables are all statistically significant and negative at the 1% level, which is generally consistent with the results in column (1). Overall, the direction of influence and significance levels of the explanatory variables did not change significantly between columns. This suggests that homestead ownership by rural migrants reduces their social integration.

**Table 3:** Regression analysis.

	Explained variable: Degree of social integration			
	(1)	(2)	(3)	(4)
Ownership of a homestead	-0.059***	-0.060***	-0.062***	-0.048***
	(0.007)	(0.007)	(0.007)	(0.007)
Gender		-0.009*	-0.010*	-0.007
		(0.006)	(0.006)	(0.006)
Age		0.018***	0.014***	0.009***
		(0.002)	(0.002)	(0.002)
Age^2^/1000		-0.160***	-0.095***	-0.060**
		(0.028)	(0.029)	(0.028)
Education		0.070***	0.064***	0.062***
		(0.003)	(0.003)	(0.003)
Nation		-0.026**	-0.031***	-0.018
		(0.011)	(0.011)	(0.011)
Political profile		0.064***	0.061***	0.060***
		(0.016)	(0.016)	(0.016)
Marriage		0.035***	-0.020**	-0.009
		(0.009)	(0.010)	(0.010)
Health		0.089***	0.090***	0.095***
		(0.007)	(0.007)	(0.007)
Monthly household income			0.021***	0.033***
			(0.007)	(0.007)
Monthly household expenditure			0.054***	0.035***
			(0.007)	(0.006)
Size of family members			0.014***	0.008***
			(0.003)	(0.003)
Duration of migration				0.012***
				(0.001)
Cities of migration				-0.003**
				(0.002)
Range of migration				-0.072***
				(0.005)
Area fe	YES	YES	YES	YES
Constant term	3.631***	2.605***	2.089***	2.367***
	(0.014)	(0.052)	(0.069)	(0.069)
Observations	68817	68817	68817	68817
R^2^	0.053	0.066	0.069	0.081

Robust standard errors in parentheses

*** p<0.01

** p<0.05

* p<0.1

### 4.2 Robustness tests

#### 4.2.1 Random sampling and variable replacement

This study uses two methods to conduct robustness tests to examine the credibility of the regression results. First, this study uses the random sampling method to test the impact of rural migrants’ homesteads on their degree of social integration. Random sampling makes each part of the overall population of survey respondents have the same probability of being sampled, and sampling was conducted in full accordance with the principle of equal opportunity. It should be noted that the random sampling method used in this study was adopted. The sampling proportions were 25% and 50%. The results of the analysis after random sampling are shown in Columns (1) and (2) of Table 4. The regression results show that the coefficients of the explanatory variables are all negatively significant at the 1% level, with only slight differences between the coefficients. Second, this section replaces the measurement dimensions of the explained variables for robustness testing. This is done by replacing the degree of social integration with "willingness to stay in the local area" as the explained variable. On this basis, the model is regressed with the same control variables and area fixed effects as in the previous section. The regression results are shown in Column (3) of Table 6. The results show that rural migrants are reluctant to settle in an area if they own homesteads. This result is consistent with the conclusions reached in the basic regression analysis, indicating that the findings of this study are robust.

**Table 4:** Robustness tests.

	Explained variable: Degree of social integration		Willingness to stay local
	25%	50%	Oprobit
	(1)	(2)	(3)
Ownership of a homestead	-0.040***	-0.042***	-0.041***
	(0.014)	(0.010)	(0.015)
Control variables	Yes	Yes	Yes
Area fe	Yes	Yes	Yes
Constant term	2.085***	2.076***	-1.796***
	(0.138)	(0.097)	(0.144)
Observations	17204	34409	68817
R^2^	0.088	0.085	-

Robust standard errors in parentheses

*** p<0.01

** p<0.05

* p<0.1

#### 4.2.2 Endogenetic test

Although this study controls for as many feature variables with as strong correlations as possible in the estimation in Table 3. However, endogenous problems arising from potential omitted variables and reverse causality, among others, should not be overlooked. However, endogeneity due to possible omitted variables and reverse causation should not be ignored. Based on the theory of Group Effect, group characteristics are closely related to a certain characteristic of an individual but do not directly affect the formation of other characteristics of an individual [40]. Therefore, the mean value of endogenous variables in a region can be used as an instrumental variable to alleviate the endogeneity problem caused by reverse causality.

Generally speaking, there is no direct correlation between the house price determined by the macro market and the degree of social integration of the migrant population. At the same time, house prices may reflect better public services and infrastructure in the city or may represent better development prospects, more suitable job opportunities, and greater room for expected wealth growth. All of these factors may affect the degree of social integration of migrant populations. Therefore, this study selects the house prices of 31 provinces (autonomous regions or municipalities directly under the Central Government) in 2017 and uses them as instrumental variables. The estimation results are shown in Column (1) of Table 5. The results of the first stage show a significant positive relationship between house prices and homestead ownership. Moreover, the F statistic value of the first stage is much larger than 10, indicating that there is no weak instrumental variable problem. Column (2) in Table 5 reports the second-stage results. The results show that the regression results are still statistically significantly negatively correlated at the 1% level after using the instrumental variables. This shows that the conclusion of this study is reliable under the premise of endogeneity control.

**Table 5:** Results of endogenous test.

	The first stage	The second stage
	ownership of a homestead	degree of social integration
	(1)	(2)
House prices	0.026***	
	(0.005)	
Ownership of a homestead		-2.102***
		(0.559)
F	242.28	-
Control variables	Yes	Yes
Area fe	Yes	Yes
Constant	0.258***	3.132***
	(0.065)	(0.304)
Observations	68,817	68,817
R^2^	0.139	-

Robust standard errors in parentheses

*** p<0.01

** p<0.05

* p<0.1

#### 4.2.3 Propensity score matching

Since the basic conditions of the rural migrant population with and without a homestead are not exactly the same, such as age, marital status, educational level, and many other factors, this may lead to certain sample self-selection problems. This may have led to biased results. Therefore, to further explore whether homestead ownership affects the integration intention of rural migrants, this study utilizes the propensity score matching (PSM) method to alleviate the estimation bias that may be caused by the sample self-selection problem.

To ensure the robustness of the propensity score matching results, this study uses the method of nearest neighbor matching (NNM) for estimation, with the goal of finding the object in the control group sample that is closest to the score of the intervention group sample to form a pair. Specifically, the explanatory variables and control variables are first regressed step-by-step to find the control variables that affect the explanatory variables. Propensity score matching was used to eliminate the influence of the control variables on the model.

Column (1) in Table 6 shows one-to-one matching, column (2) shows one-to-two matching, column (3) shows one-to-three matching, column (4) shows one-to-four matching, and column (5) shows one-to-five matching. The regression results show that all five matching methods are significant at the 1% level. The estimation results of the five different matching methods are consistent, indicating that the PSM results are robust. Further found that owning, homestead ownership has a significant inhibitory effect on the degree of social integration of rural migrants.

**Table 6:** Results of propensity score matching.

	Explained variable: Degree of social integration				
	(1)	(2)	(3)	(4)	(5)
Ownership of a homestead	-0.044***	-0.049***	-0.052***	-0.049***	-0.047***
	(0.011)	(0.009)	(0.009)	(0.008)	(0.008)
Control variables	Yes	Yes	Yes	Yes	Yes
Area fe	Yes	Yes	Yes	Yes	Yes
Constant term	2.718***	2.541***	2.568***	2.578***	2.566***
	(0.185)	(0.169)	(0.162)	(0.158)	(0.158)
Observations	23,258	33,633	40,134	44,920	48,539
R^2^	0.090	0.090	0.089	0.089	0.088

Robust standard errors in parentheses

*** p<0.01

** p<0.05

* p<0.1

#### 4.2.4 Placebo test

**Fig 1:**
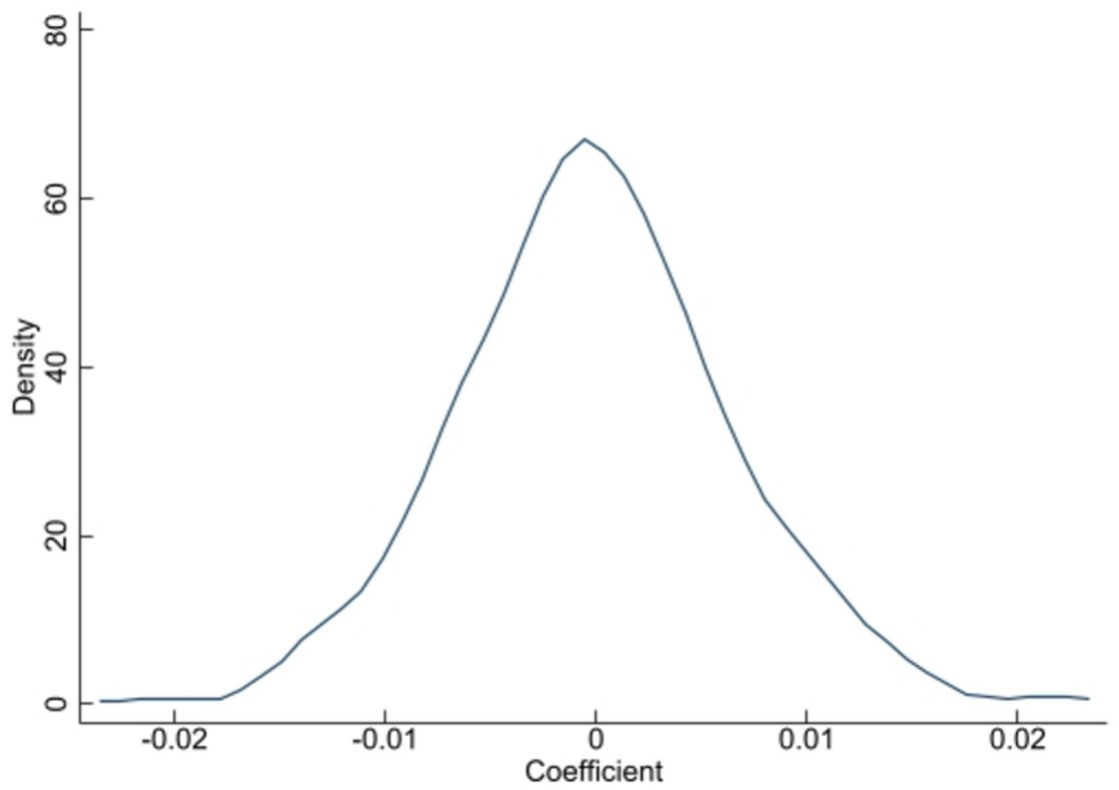
Placebo test.

The level of economic development varies greatly among the regions in China. Although we included regional dummy variables to fix the regions, it is still possible that these characteristics may have different impacts on the study groups. Therefore, to further verify the robustness of the model, this study utilizes a placebo test to demonstrate that the differences in the integration intentions of rural migrants are caused by the influence of owning a homestead. This is done by first randomly disrupting the order of the explanatory variables and placing them into a repeated regression in Model (1) to produce an estimated coefficient $\hat{\beta }$. This process was repeated 500 times, resulting in a value of 500 $\hat{\beta }$. Fig 1 shows the distribution of $\hat{\beta }$. It can be observed from the figure that the distribution of $\hat{\beta }$ is close to the standard normal distribution with a mean close to 0. This indicates that the estimated equation passed the placebo test [41].

## 5. Heterogeneity tests

### 5.1 Heterogeneity tests at the age level

Individuals’ economic behavior during their life cycle changes with age [42]. There may be large differences in housing needs, ideology, and family responsibilities among different age groups. Therefore, this study examines the effect of rural migrants of different age groups on their social integration from an age-generational perspective. Based on the existing research, this study divides the sample into three sub-samples of 18–35 years old, 36–50 years old and over 50 years old for regression, and the regression results are shown in Table 7. For the rural migrant population aged 18 to 50, owning a homestead has a significant negative impact on the degree of social integration in the city of inflow. However, it is not significant for rural migrants over 50 years old.

**Table 7:** Heterogeneity effects based on age.

	Explained variable: Degree of social integration		
	18–35	35–50	>50
	(1)	(2)	(3)
Ownership of a homestead	-0.050***	-0.045***	-0.041
	(0.014)	(0.016)	(0.033)
Control variables	Yes	Yes	Yes
Area fe	Yes	Yes	Yes
Constant term	2.289***	3.019***	1.424
	(0.221)	(0.572)	(1.995)
Observations	35,157	27,854	5,806
R^2^	0.086	0.078	0.079

Robust standard errors in parentheses

*** p<0.01

** p<0.05

* p<0.1

### 5.2 Heterogeneity tests at the regional level

There are differences in the level of economic development and access to public services between different regions. Therefore, we ask the question: What is the impact of homestead ownership by rural migrants in different regions on their degree of social integration? In response to this question, this section concludes the paper. The regression results are presented in Table 8. In the table, it can be found that only the regression coefficient of the central region is not significant. This indicates that homestead ownership by rural migrants in the central region does not affect their integration in the city. However, the western and eastern regions were statistically significant at the 5% and 10% levels, respectively. For the eastern regions, a better level of economic development also means that the competition for jobs as well as the pressure in terms of life are higher in these places. When people are under more pressure, they devote their limited energy to maintaining their lives and will have less or no emotional resonance with the city of their inflow. Therefore, when homesteads can provide support for rural migrants in the eastern region, it will be more difficult for them to part with their attachment to their hometowns, making them less willing to integrate into the cities of their inflow areas. As for rural migrants in the western region, their development situation is average or even backward. Therefore, even if the rural migrant population in this region flows to the city, their sense of belonging to the city of inflow will not be too strong, and this effect will be even stronger when they have homesteads.

**Table 8:** Heterogeneity effects based on region.

	Explained variable: Degree of social integration		
	western region	central region	Eastern region
	(1)	(2)	(3)
Ownership of a homestead	-0.068**	-0.030	-0.040*
	(0.028)	(0.020)	(0.018)
Control variables	Yes	Yes	Yes
Area fe	Yes	Yes	Yes
Constant term	2.777***	2.995***	1.789***
	(0.308)	(0.141)	(0.166)
Observations	17035	16986	34796
R^2^	0.061	0.052	0.095

Robust standard errors in parentheses

*** p<0.01

** p<0.05

* p<0.1

## 6. Mechanisms test and further analysis

Fundamentally, housing is an essential part of people’s livelihood. As long as the local housing problems of rural migrants are resolved, they will be able to live and work in peace and contentment. Only in this way can the rural migrant population’s sense of integration and belonging be enhanced. It has been confirmed that the higher the housing expenditure of the migrant population in the city, the more it inhibits the degree of social integration of the rural migrant population. As mentioned in the previous theoretical analysis section, there is also "resistance" beyond the combined effect of push and pull, which affects population mobility and social integration. Among the sample selected in this study, 66.48% of the respondents believed that not being able to afford a house was the main difficulty in living locally, which shows that the housing problem has become a kind of resistance that affects the social integration of rural migrants in the cities of influx. Table 9 reports the regression results for impact mechanisms [43]. The results in Column (2) indicate that housing expenditure inhibits the level of social integration of rural migrants, and the results are consistent with the above analysis. The results in column (3) show that the coefficient estimates for both housing expenditure and homesteads are negative and remain statistically significant at the 1% level. This indicates that the higher the housing expenditure, the lower is the degree of social integration of the rural migrant population.

**Table 9:** Mechanism test.

	Explained variable: Housing expenditure	Explained variable: Degree of social integration	
	(1)	(2)	(3)
Ownership of a homestead	0.187***		-0.047***
	(0.027)		(0.007)
Housing expenditure	-	-0.005***	-0.005***
		(0.001)	(0.001)
Control variables	Yes	Yes	Yes
Area fe	Yes	Yes	Yes
Constant term	-7.711***	2.307***	2.328***
	(0.265)	(0.069)	(0.069)
Observations	68,817	68,817	68,817
R^2^	0.136	0.081	0.081

Robust standard errors in parentheses

*** p<0.01

** p<0.05

* p<0.1

Rural contracted land and homesteads belong to two categories of material elements. Rural contracted land is a means of production, while homesteads are the means of living. Current land contract law can protect rural residents’ rights to contract land management. Land management law also follows rural residents’ willingness to voluntarily withdraw from homesteads with compensation. Therefore, the rural population with contracted land and homesteads will have psychological "backing" support after flowing into other cities. This kind of "backing" support will also limit the influence of this kind of group on the degree of social integration in the city of inflow to a certain extent. At the same time, if the migrant population owns their own housing in the city, this will also affect their social integration in the city of influx.

Based on this, this paper further incorporates "whether or not they own contracted land" and "whether or not they own their own home" into the model for analysis. The results in column (1) of Table 10 show that the coefficient of the interaction term (Treat1) is negative and significant at the 10% level of statistical significance. This result suggests that if rural migrants also own contracted land, their social integration is further reduced. The result in column (2) shows that the coefficient of the interaction term (Treat2) is positive, and the coefficient is statistically significant at the 10% level. A possible explanation is that the group that owns a house in the city of inflow perceives themselves as more localized than the group that does not own a house. In other words, these groups subjectively achieved social integration.

**Table 10:** Further analysis.

	Explained variable: Degree of social integration	
	(1)	(2)
Treat1	-0.025*	
	(0.015)	
Ownership of a contracted land	0.033**	
	(0.013)	
Treat2		0.029*
		(0.016)
Ownership of a house		0.121***
		(0.013)
Ownership of a homestead	-0.044***	-0.045***
	(0.009)	(0.008)
Control variables	Yes	Yes
Area fe	Yes	Yes
Constant term	2.362***	2.568***
	(0.069)	(0.070)
Observations	68,817	68,817
R^2^	0.081	0.085

Robust standard errors in parentheses

*** p<0.01

** p<0.05

* p<0.1

## 7. Conclusions and recommendations

### 7.1 Conclusions

This study systematically explores the impact of homesteads on the social integration of rural migrant populations and their mechanism of action by utilizing the 2017 China Migrants Dynamic Survey and matched urban macro data. The study shows that homestead ownership by the rural migrant population inhibits the degree of social integration. This effect varies with factors, such as individual and regional characteristics. Heterogeneity analysis shows that when rural migrants between the ages of 18 and 50 hold a homestead, their integration into the city of inflow is inhibited. However, for rural migrants over 50 years of age, the presence or absence of homesteads does not affect their degree of social integration. At the same time, when rural migrants in the central region own homesteads, their social integration in the city of inflow is not affected. The mechanism analysis shows that rural migrants with homesteads inhibit social integration by increasing their housing expenditures. Further analysis shows that the ownership of contracted land and self-owned housing affects the level of homestead and social integration of rural migrants. The conclusions drawn in this study can provide theoretical references for improving the degree of social integration of the migrant population, enhancing people’s well-being, and improving the mechanism of urban-rural integration and development.

### 7.2 Recommendations

The above findings provide empirical evidence for advancing the urbanization process and social integration of the migrant population. Based on this, the study proposes the following recommendations.

First, the government should improve and perfect the homestead withdrawal system. Specifically, the government should focus on encouraging and supporting the voluntary withdrawal of property rights holders from vacant homesteads, fully respecting people’s wishes and demands. At the same time, it should continue to standardize the withdrawal mechanism of rural residential bases to enhance the willingness of the rural migrant population to stay in the city. Only in this way can the urbanization process be better promoted. In addition, the government should strengthen the organization and reclamation of vacant homesteads, thus improving the efficiency of rural land use. Of course, it is also very important to improve the rights and interests protection mechanism of the population withdrawing from the residential base. For example, the relevant government should fully respect the wishes of the rural migrant population when encouraging or supporting homestead withdrawal. By safeguarding the land rights and interests of these groups, the fruits of urbanization can be shared.

Second, when formulating policies, the government should fully consider the individual characteristics and preferences of the farmers. Policies should be formulated for farmers with different characteristics. Specifically, the government should prioritize the construction of incentive mechanisms for the withdrawal of residential land for special groups such as "empty-nesters" in rural areas. Simultaneously, a long-term mechanism to promote stable employment for young and middle-aged migrants can also be established. It is also important to strengthen vocational training for the migrant population, increase the number of jobs in cities and towns, and raise the economic income of the migrant population. Only through these measures can the migrant population’s willingness to stay in the city and the degree of social integration be improved.

In addition, regions with different levels of economic development have different economic levels, educational resources, infrastructure construction, and other aspects. Therefore, the willingness of the migrant population in different regions to stay in the city also shows the characteristics of travel alienation. In this regard, differences in the development of various regions should be fully considered in the process of promoting urbanization and citizenship, and targeted policies should be formulated. The corresponding policy system should be improved in terms of employment support, residential security, and public services to enhance the attractiveness of cities to the floating population.

Finally, the government must establish a sound housing security system. Specifically, more preferential policy support will be provided to farmers who voluntarily withdraw from homesteads, such as public rental housing, resettlement housing, and commercial housing purchases. Simultaneously, financial support should continuously increase. Specifically, the coverage of housing provident funds can be continuously expanded to alleviate housing pressure on the rural migrant population in cities, thereby enhancing the migrant population’s willingness to stay in the cities. Of course, community building must be strengthened. For example, it is necessary to establish a culture that aims to build a harmonious community and promote social integration. At the same time, it is very important to actively carry out all kinds of community activities that can strengthen the interaction between the migrant population and local citizens. Through such interactions, integration between the migrant population and local citizens can be strengthened, thereby enhancing the migrant population’s sense of belonging. A higher sense of belonging makes the migrant population more willing to stay in the city, thus promoting the process of urbanization.

### 7.3 Limitations

This study still has the following limitations:

Firstly, the research relies on cross-sectional data from the 2017 China Migrants Dynamic Survey, which limits the ability to establish causal relationships between homestead ownership and social integration. Longitudinal data could provide a more robust understanding of the dynamics over time.

Secondly, the study’s focus on rural migrant populations in China may restrict the generalizability of the findings to other contexts with different migration patterns or socioeconomic conditions. Future research could explore how homestead ownership affects social integration in diverse global settings.

Lastly, while the study identifies the inhibitory effects of homestead ownership on social integration, it may overlook potential positive aspects or cultural meanings attached to homestead ownership among rural migrants. Further research could delve into the nuanced complexities surrounding homesteads and social integration.

Despite these limitations, the conclusions drawn in this study offer valuable theoretical references for policymakers, urban planners, and researchers seeking to address the challenges faced by migrant populations and promote social integration in urban settings.

## Supporting information

S1 Data (XLSX)
